# Experimental study on the reasonable proportions of rock-like materials for water-induced strength degradation in rock slope model test

**DOI:** 10.1038/s41598-023-36511-8

**Published:** 2023-06-07

**Authors:** Yuan Cui, Chao Xu, Lei Xue, Jinyu Dong, Tong Jiang

**Affiliations:** 1 Key Laboratory of Shale Gas and Geoengineering, Institute of Geology and Geophysics, Chinese Academy of Sciences, Beijing, 100029 China; 2 Innovation Academy for Earth Science, Chinese Academy of Sciences, Beijing, 100029 China; 3 College of Earth and Planetary Sciences, University of Chinese Academy of Sciences, Beijing, 100049 China; 4 College of Geoscience and Engineering, North China University of Water Resources and Electric Power, Zhengzhou, 450045 China

**Keywords:** Natural hazards, Engineering, Materials science

## Abstract

Water-induced strength deterioration of rock mass is a crucial factor for rock slope instability. To better show the degradation process of rock slope water–rock interaction, we used bentonite as a water-sensitive regulator to build a new rock-like material that matches the features of water-induced strength degradation based on the cement-gypsum bonded materials. Twenty-five schemes of the material mixture proportion were designed using the orthogonal design method considering four factors with five variable levels, and a variety of experiments were conducted to obtain physico-mechanical parameters. In addition, one group of rock-like material proportion was selected and applied to the large-scale physical model test. The experiment results reveal that: (1) The failure mode of this rock-like material is highly similar to that of natural rock masses, and the physico-mechanical parameters vary over a wide range; (2) The bentonite content has a significant influence on the density, elastic modulus, and tensile strength of rock-like materials; (3) It is feasible to obtain the regression equation based on the linear regression analysis to determine the proportion of rock-like material; (4) Through application, the new rock-like material can effectively simulate or reveal the startup mechanism and instability characteristics of rock slopes under water-induced degradation. These studies can serve as a guide for the fabrication of rock-like material in the other model tests.

## Introduction

Slope instability often causes huge economic losses and casualties, so the mechanism of slope instability has always been a frontier issue in the international research of engineering geology^1,2^. The weakening effects caused by water–rock interaction significantly impact on the stability of rock slopes^3,4^. Numerous engineering studies have shown that slope instability is typically observed during the wet season^5–7^. Large-scale rock landslides are usually challenging to examine due to their complicated structure, strong suddenness, and strong concealment^8,9^. The physical model test can effectively reveal the deformation and evolution laws of rock masses under complex geological conditions in rock engineering, which has the advantages of high operability, a short cycle, and intuitive results^10–13^. Because the materials and amount of mixing have a significant impact on the properties of the physical model, a scientific and acceptable selection of similar material is the prerequisite of the model test^14,15^.

Since the development of physical model tests, a great deal of study has been undertaken on the proportion of rock-like materials. Numerous preliminary researches were conducted to discover the fundamental principles of selecting and mixing of rock-like materials^16–18^. Subsequently, an increasing number of materials were employed to imitate different types of rocks, which could be categorized into three groups: hard rock, soft rock, and fluid–solid coupling materials. Physico-mechanical properties, including strength and brittleness, are the primary indicators of resemblance for hard rock-like materials. The cement-gypsum bonded material is the most commonly used to simulate hard rock, and its mechanical behaviors are consistent with those of natural rock mass. However, its curing period is longer^19–21^. The creation of rosin and alcohol-like materials has drastically reduced the curing time of rock-like materials, and these materials also boast a high volumetric weight, low elastic modulus, and stable performance. However, the brittleness of it will be reduced at the same time^22–24^. For soft rock-like materials, its softening effect should be considered to improve the validity of model tests^25–27^. The aforementioned rock-like material greatly enriched the research of rock-like materials and laid the foundation for future model test research.

**Figure 1:**
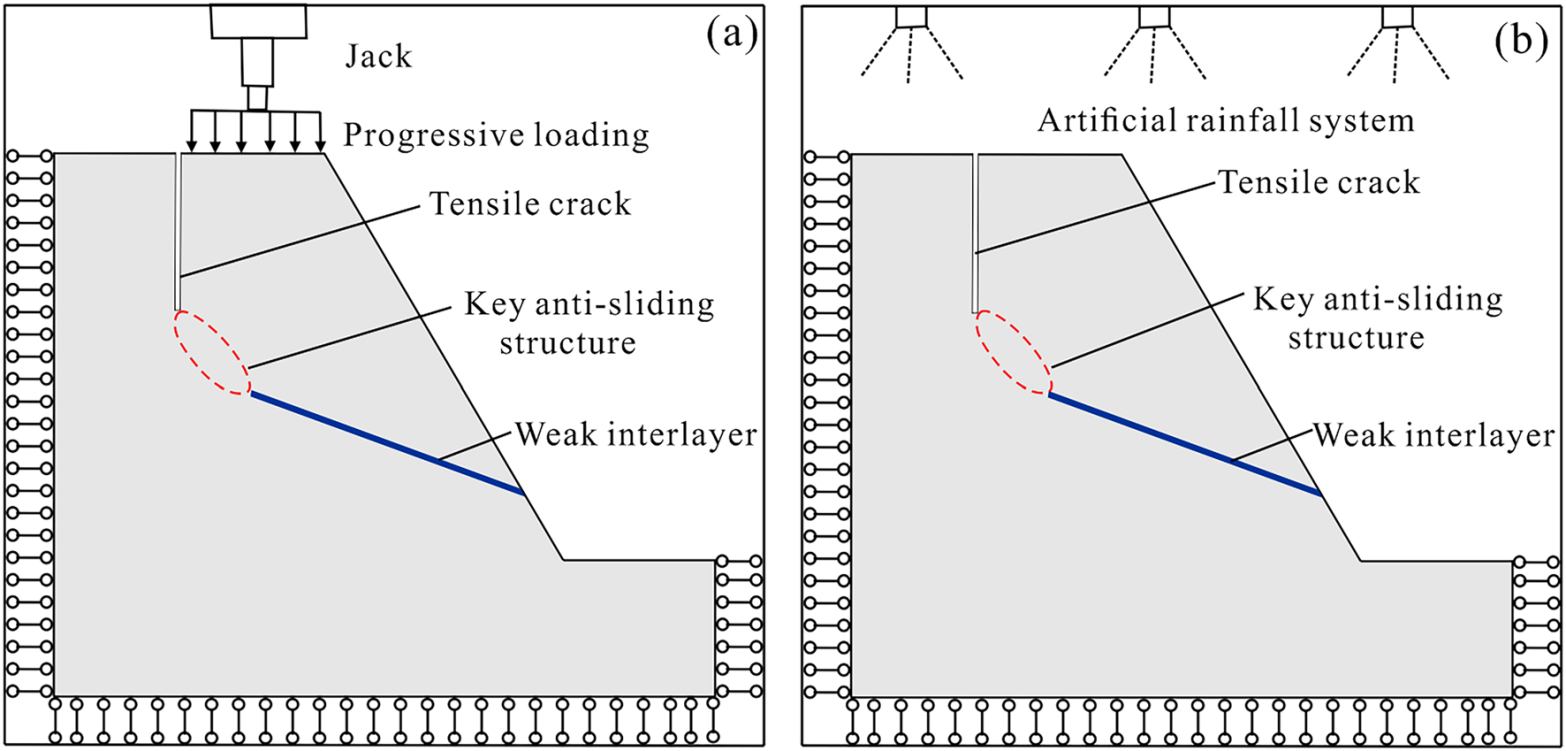
The main ways to facilitate the slope failure in the physical model tests. (**a**) A jack with progressive loading; (**b**) The artificial rainfall.

Obviously, the previous researches on rock-like materials concentrated mostly on the similarity of their physico-mechanical properties, whereas there are few attentions on the disintegrating properties of brittle rock masses under water–rock interaction. In actual geological engineering, such as large-scale rock landslides that occur during rainfall, which instabilities were mainly triggered by the strength degradation of the essential anti-sliding structure under water–rock interaction^28^. For such conditions, the method of applying a jack with progressive loading to facilitate slope failure cannot consider the degradation effect of water on the rock^29,30^ (Fig. 1a). In addition, using artificial rainfall is an effective way to simulate hydraulic damage, which, yet, is commonly suitable for rainfall-induced soil landslides^31,32^. For rock slopes, the strength degradation of conventional rock-like materials responding to the action of artificial rainfall is imperceptible, and the precise monitoring equipment is prone to be broken during the rainfall process (Fig. 1b). Thus, it is of great urgency to design a rock-like material that can efficiently reappear the strength loss of rock due to the water–rock interaction.

This work aims to design a new rock-like material with high bulk weight and water sensitivity for used in model test studies of water-induced degradation of large rock slopes. Based on the traditional cement-gypsum bonded material, bentonite was added to regulate the water sensitivity of the material. Using the orthogonal experiment method, 25 schemes of the material mixture proportion of rock-like materials with different proportions were designed. Subsequently, the range analysis and variance analysis were performed to reveal the significance of various factors on the physico-mechanical parameters of rock-like materials, and quantified the correlation between influencing factors and physico-mechanical parameters. Finally, a physical model of the rock slope was built with rock-like materials, and the water-induced degradation of the slope was realized by water injection. The startup mechanism and instability characteristics of rock slopes under water-induced degradation were investigated. The results of this study overcome the shortcomings of the traditional hard rock materials that are inapparent in disintegrating properties and serve as a reference for determining the proportion of rock-like materials in subsequent large-scale rock landslide model tests.

## Materials and methods

### Experimental scheme

The orthogonal design is an effective way for studying problems with multiple factors and levels. On the basis of orthogonality, which is characterized by balanced dispersion, comparability, and uniformity, it picks representative experiments from the entire experimental scheme^33^.

Regarding previous research results^34^, barite powder (200 mesh), coarse sand (40–70 mesh), fine sand (20–40 mesh), and bentonite (400 mesh) were selected as aggregates, while cement (42.5 grade) and gypsum (120 mesh) were selected as cementing materials in this study. As shown in Table 1, where aggregate-binder ratio is the ratio of aggregate to binder and cement-gypsum ratio is the ratio of cement to gypsum. This experiment utilized the orthogonal design scheme L_25_ (56) with four factors and five levels. Table 2 outlines the experimental scheme.

**Table 1:** Selection of factors and levels for the orthogonal experiment.

Level	Aggregate-binder ratio (A)	Cement-gypsum ratio (B)	Barite content (C)	Bentonite content (D)
1	4:1	3:7	0.15	0.00
2	5:1	4:6	0.20	0.10
3	6:1	5:5	0.25	0.20
4	7:1	6:4	0.30	0.30
5	8:1	7:3	0.35	0.40

**Table 2:** Experimental schemes of rock-like material.

Group number	Aggregate-binder ratio (A)	Cement-gypsum ratio (B)	Barite content (C)	Bentonite content (D)
1	4:1	3:7	0.15	0.00
2	4:1	4:6	0.20	0.10
3	4:1	5:5	0.25	0.20
4	4:1	6:4	0.30	0.30
5	4:1	7:3	0.35	0.40
6	5:1	3:7	0.20	0.20
7	5:1	4:6	0.25	0.30
8	5:1	5:5	0.30	0.40
9	5:1	6:4	0.35	0.00
10	5:1	7:3	0.15	0.10
11	6:1	3:7	0.25	0.40
12	6:1	4:6	0.30	0.00
13	6:1	5:5	0.35	0.10
14	6:1	6:4	0.15	0.20
15	6:1	7:3	0.20	0.30
16	7:1	3:7	0.30	0.10
17	7:1	4:6	0.35	0.20
18	7:1	5:5	0.15	0.30
19	7:1	6:4	0.20	0.40
20	7:1	7:3	0.25	0.00
21	8:1	3:7	0.35	0.30
22	8:1	4:6	0.15	0.40
23	8:1	5:5	0.20	0.00
24	8:1	6:4	0.25	0.10
25	8:1	7:3	0.30	0.20

### Production process

**Figure 2:**
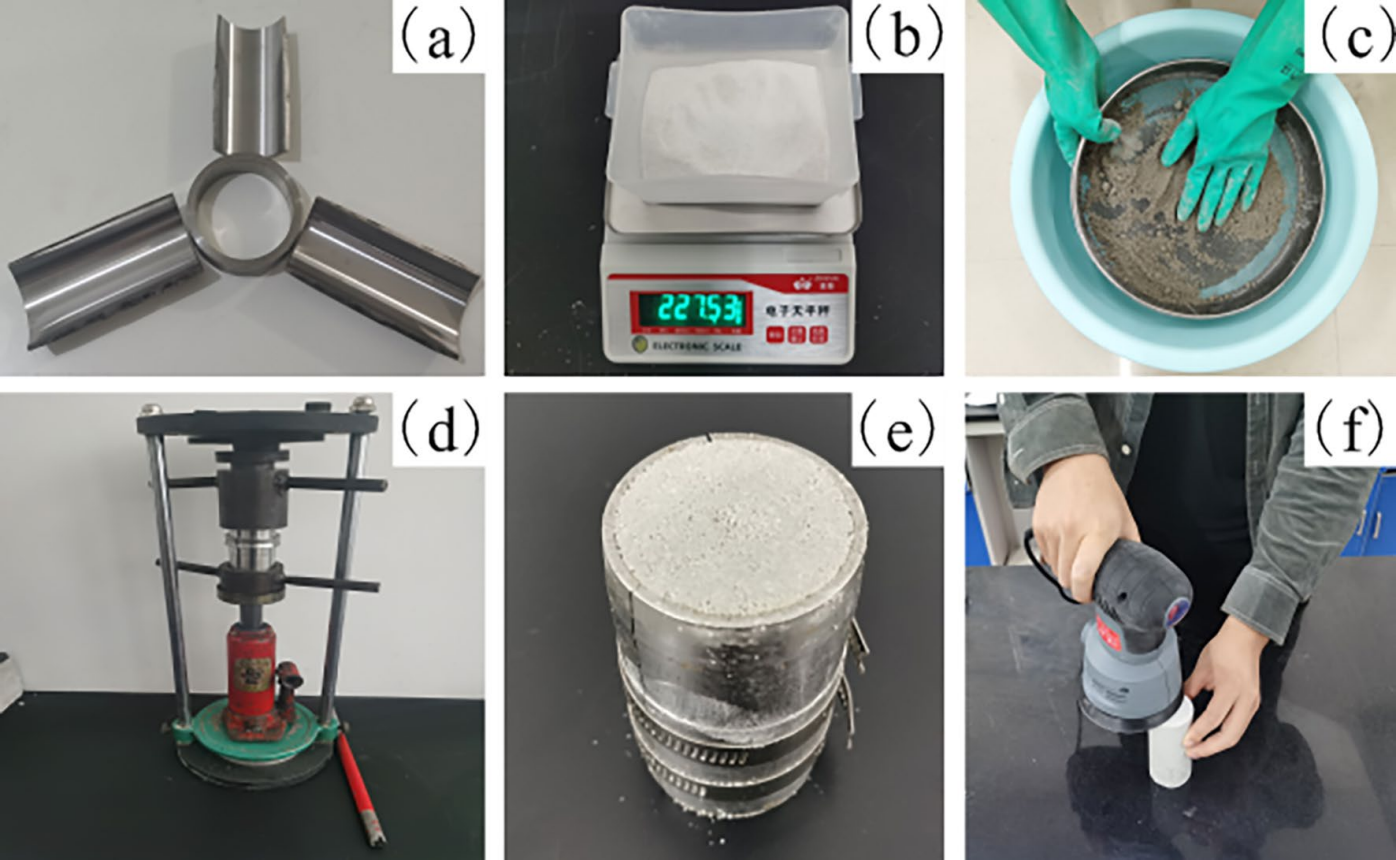
The fabrication process of the rock-like material sample. (**a**) Three-petal steel mold, (**b**) weighing, (**c**) stirring, (**d**) compacting, (**e**) demolding, and (**f**) polishing.

The standard cylindrical samples of rock-like material were produced using a standard three-petal steel mold (Fig. 2a). After assembling the mold, the sample was created by following the procedures of weighing (Fig. 2b), stirring (Fig. 2c), compacting (Fig. 2d), demolding (Fig. 2e), and polishing (Fig. 2f).

**Figure 3:**
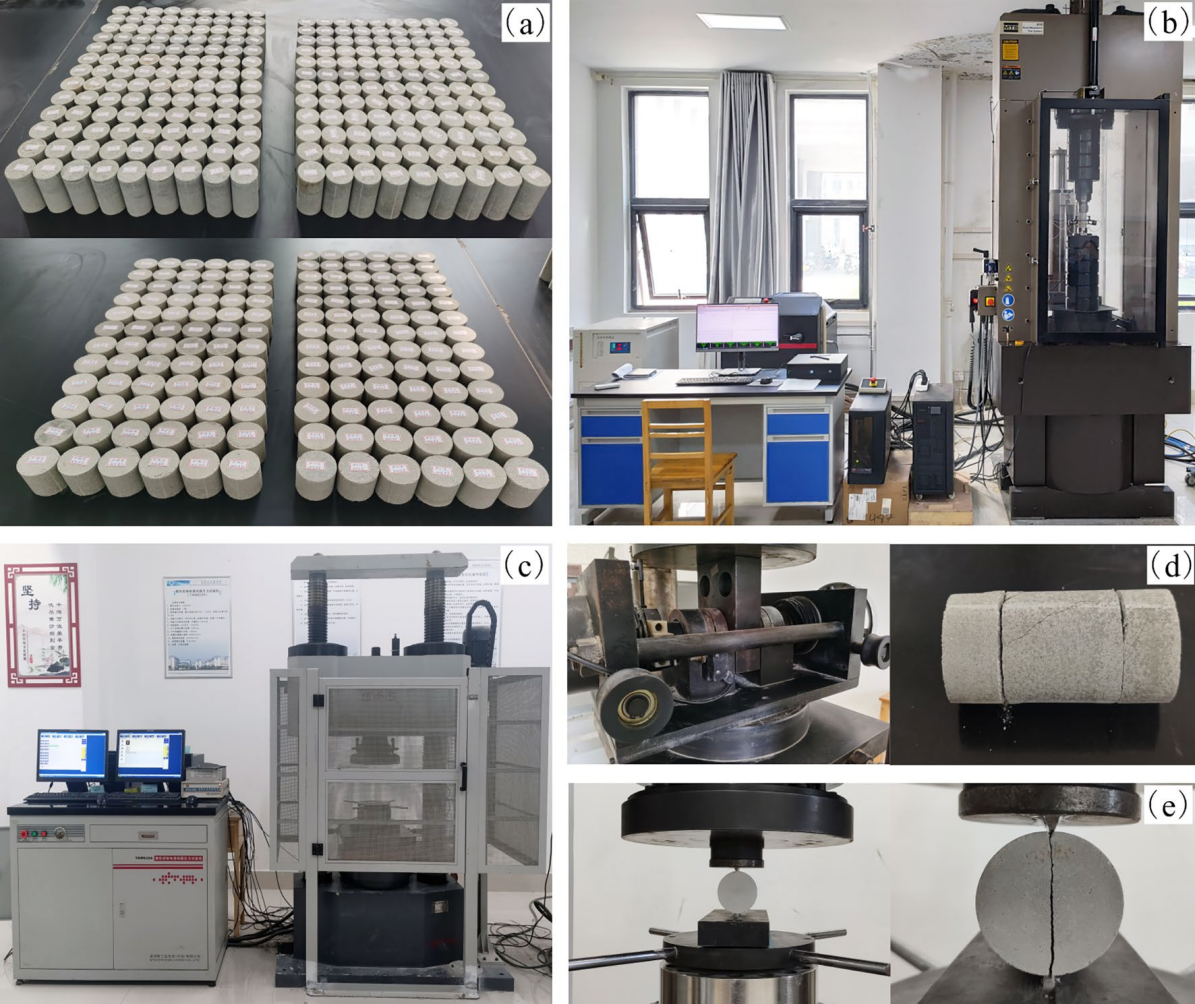
Mechanical parameter tests of samples. (**a**) Experimental samples; (**b**) MTS-815 rock mechanics testing machine; (**c**) YAW6206 computer-controlled electro-hydraulic servo pressure testing machine; and (**d**) direct shear test; (**e**) split test.

To determine the physico-mechanical parameters of rock-like material, uniaxial compression tests, split tests, and direct shear tests were carried out on the samples. A total of 375 samples were produced, of which 155 (Fig. 3a) were used for the uniaxial compression test with an MTS-815 rock mechanics testing machine (Fig. 3b), and other samples were used for the split test and shear test with the YAW6206 computer-controlled electro-hydraulic servo pressure testing machine (Fig. 3c). Figure 3d and e depict the failure modes of direct shear and split samples, respectively.

## Results and analysis

**Figure 4:**
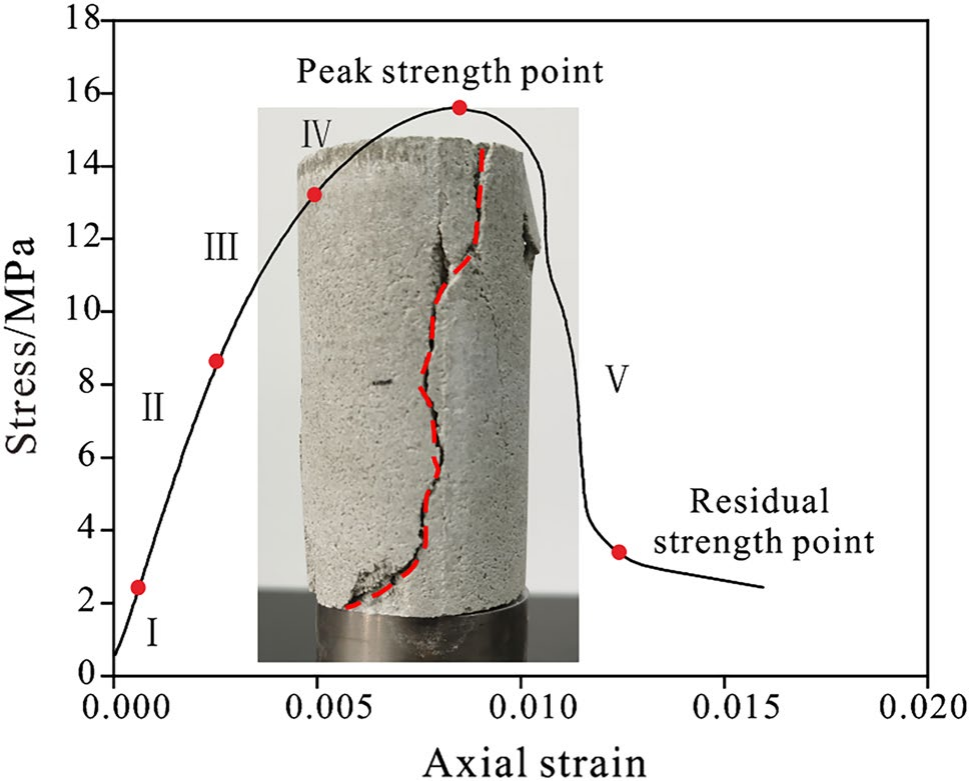
The uniaxial compression stress–strain curve of sample.

As depicted in Fig. 4, the stress–strain curve of sample exhibits five distinct stages: the crack closure stage (I), the elastic deformation stage (II), the stable rupture stage (III), the unsteady rupture stage (IV) and the post-peak stage (V), which demonstrates excellent elasticity and plasticity. In addition, the failure forms were predominantly tensile fracture failure and diagonal shear failure, which are highly similar to the typical failure characteristics of real rock masses, and can more accurately reflect their mechanical properties.

To prevent the dispersion of measured data, four specimens were prepared for each group such that at least two results were consistent. The flatness and perpendicularity tolerances of the specimen met the ISRM requirements^35^. 25 groups of different proportion material samples were tested for physico-mechanical properties (Table 3).

**Table 3:** Experiment results of rock-like material proportion.

Group number	Density/(g/cm^3^)	UCS/MPa	Elastic modulus/GPa	Poisson’s ratio	Tensile strength/MPa	Internal friction angle/(°)	Cohesion/MPa
1	2.12	17.17	5.68	0.16	1.28	60.25	4.03
2	2.16	23.66	5.04	0.17	1.83	51.59	4.80
3	2.10	25.55	4.60	0.16	1.70	33.18	8.94
4	2.04	22.54	3.94	0.18	1.79	52.18	6.49
5	1.98	22.36	3.86	0.18	1.71	36.36	4.15
6	2.09	17.07	2.42	0.15	1.53	46.00	6.12
7	2.03	15.32	2.32	0.15	1.24	38.40	6.00
8	2.00	20.94	2.78	0.16	1.34	46.35	5.85
9	2.08	14.70	5.01	0.17	1.28	52.20	4.52
10	2.18	21.33	5.75	0.17	2.46	53.41	3.77
11	1.95	14.23	1.90	0.13	0.91	42.86	3.10
12	2.10	17.87	4.46	0.15	1.20	45.60	4.58
13	2.09	17.49	4.10	0.17	1.98	53.27	4.87
14	2.14	16.99	3.34	0.17	1.66	54.66	4.60
15	2.05	22.46	3.79	0.16	1.59	45.90	4.75
16	2.10	13.52	2.31	0.13	1.85	39.98	4.93
17	2.04	15.65	3.26	0.16	1.45	46.59	3.67
18	2.05	15.50	2.31	0.13	1.17	48.43	2.95
19	2.03	16.70	2.49	0.14	1.28	38.47	4.97
20	2.08	13.72	3.92	0.16	1.00	46.51	4.05
21	1.96	10.38	1.66	0.13	0.96	35.84	2.81
22	1.98	10.88	1.63	0.10	0.97	32.74	2.95
23	2.02	7.24	2.88	0.15	1.06	47.41	2.09
24	2.14	15.44	3.95	0.16	1.82	38.72	4.77
25	2.06	15.08	2.63	0.17	1.70	52.93	3.42

The density distribution of rock-like material ranges from 1.95 to 2.18 g/cm^3^, indicating that this material has a relatively high bulk density. The UCS is distributed in the range of 7.24–25.55 MPa, the elastic modulus is distributed in the range of 1.63–5.75 GPa, the Poisson's ratio is distributed within 0.14–0.18, the tensile strength is distributed in 0.91–2.46 MPa, the internal friction angle is distributed in the range of 32.74–60.25°, and the cohesion is distributed in the 2.09–8.94 MPa, indicating that the mechanical parameters of the rock-like material have a large adjustable range, which can meet the requirements of most rock mass model tests for rock-like materials.

### Density sensitivity analysis

Range analyses and analysis of variance were used to determine the sensitivity and significance of the four factors in orthogonal experiment schemes to different physico-mechanical parameters of rock-like materials. Range analysis can intuitively distinguish the primary and secondary factors of the experiment, whereas the analysis of variance is a widely used statistical test that analyzes differences and significance among multiple groups samples^33^.

**Figure 5:**
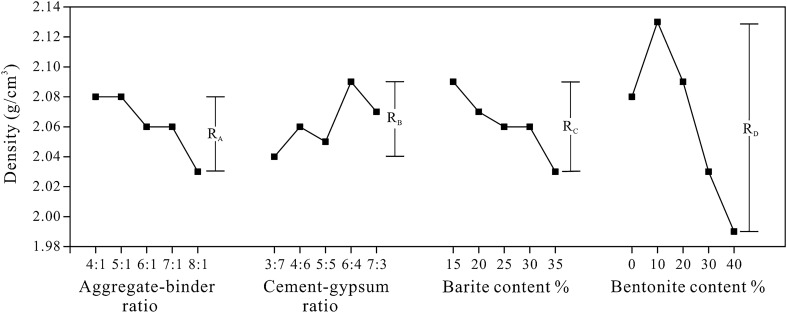
Sensitivity analysis of the density.

Figure 5 depicts the sensitivity analysis of different factors on density. The ‘R’ represents range, and the subscript A-D corresponds to the factor A-D respectively. According to the extremum difference of density (R-value), the bentonite content is the most sensitive factor to density, and other factors have a similar degree of influence, indicating that the bentonite content plays a significant role in determining the density of rock-like materials. As illustrated in Fig. 5, as the aggregate-binder ratio and barite content increase, the density reduces dramatically. The reason for this is that the content of quartz sand drops as barite powder increases, which has a significant impact on density. With an increase in bentonite content (i.e. from 0 to 40%), the sample density first reaches a maximum (i.e. around 2.13 g/cm^3^) and then drops to less than 2 g/cm^3^. This is due to the fact that when the content of bentonite remaining is low, bentonite particles with a smaller diameter will fill the gap between coarse aggregates, hence increasing sample compaction and density. However, bentonite has a lower apparent density than quartz sand and barite powder.

Table 4 shows the variance analysis of density. In general, p < 0.05 indicates that the factor has a significant impact on the physico-mechanical parameter of the materials, furthermore p < 0.01 indicates that the effect is quite significant. The greater the F value and the lower the p-value, indicating a more reliable result. The results of the variance analysis prove that bentonite content has a significant effect on the density of rock-like materials. Consistent with the findings of the sensitivity analysis, the remaining parameters had little effect on density.

**Table 4:** The variance analysis of the density of rock-like material.

Variance sources	Seq SS	df	MS	F	p-value	Significant
A	0.00676	4	0.00169	0.41748	0.79406	No
B	0.00541	4	0.00135	0.32836	0.85561	No
C	0.00956	4	0.00239	0.61085	0.65961	No
D	0.06288	4	0.01572	12.63648	0.00003	Yes
Error	0.08776	20				

### UCS sensitivity analysis

**Figure 6:**
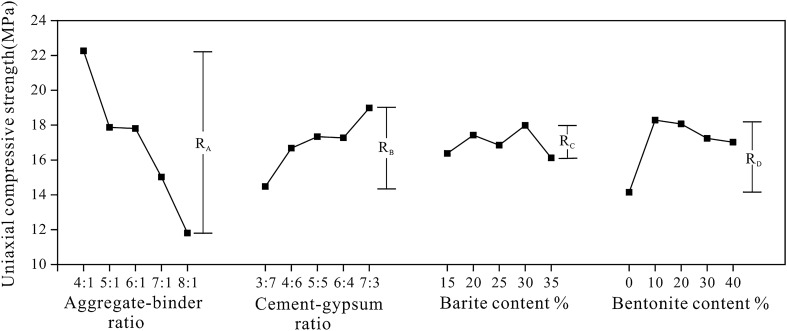
Sensitivity analysis of the UCS.

Figure 6 depicts the sensitivity analysis of different factors on UCS. According to the R-value, the most sensitive factor to UCS is the aggregate-binder ratio, which rose from 4:1 to 8:1, resulting in a 46.9% decrease in UCS of rock-like material. Other factors have a similar degree of impact, demonstrating that the aggregate-binder ratio is the most important factor in determining the UCS of rock-like materials. As demonstrated in Fig. 6, the UCS increases dramatically as the aggregate-binder ratio decreases and the cement-gypsum ratio increases. The former is because, when the aggregate-binder ratio grows, the cementing material content drops, and the bonding ability of the sample is decreased, resulting in a reduction of strength. The latter is because cement can increase the strength of the material as a hydraulic cementing material^36^. The UCS of the rock-like material is significantly improved when the content of bentonite is increased from 0 to 10%. This is mostly because bentonite fills the spaces between the quartz sand particles, which causes the UCS increase with the increase of the compactness of the sample. The UCS of the rock-like material tends to decrease with the continuous increase of bentonite content. This is due to the fact that when the bentonite content of the sample increases, the cementation degree of the sample weakens, reducing the compressive strength of the sample.

The variance analysis for UCS is displayed in Table 5. Consistent with the findings of the sensitivity analysis, the results indicate that the aggregate-binder ratio has a substantial effect on the UCS of rock-like materials, whereas other factors are insignificant when the parameter level changes.

**Table 5:** The variance analysis of the UCS of rock-like material.

Variance sources	Seq SS	df	MS	F	p-value	Significant
A	299.6698	4	74.91745	8.939	0.0003	Yes
B	53.0956	4	13.27390	0.641	0.6395	No
C	11.7033	4	2.925831	0.128	0.9703	No
D	55.1822	4	13.79555	0.670	0.6207	No
Error	467.2930	20				

### Elastic modulus sensitivity analysis

**Figure 7:**
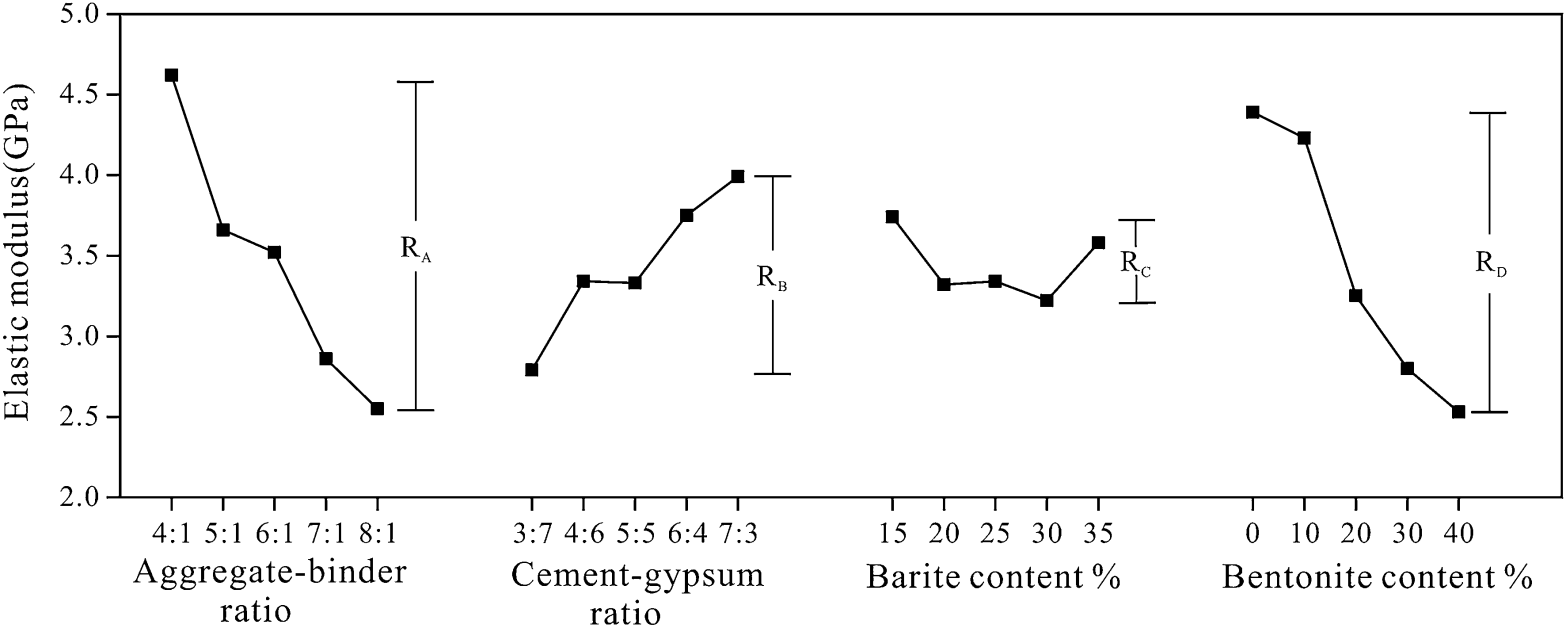
Sensitivity analysis of the elastic modulus.

Figure 7 depicts the sensitivity analysis of different factors on elastic modulus. According to R-value, the most sensitive factor is the aggregate-binder ratio, which increased from 4:1 to 8:1 while the elastic modulus of rock-like material decreased by 45.02%. Other factors have a similar degree of impact, demonstrating that the aggregate-binder ratio is the most significant factor in determining the elastic modulus of rock-like material. And the elastic modulus reduces dramatically with an increase in aggregate-binder ratio and bentonite content.

Table 6 shows the variance analysis of elastic modulus. Consistent with the results of the sensitivity analysis, the variance analysis indicates that the aggregate-binder ratio and the bentonite content have a substantial effect on the elastic modulus of rock-like materials, whereas other factors are unimportant when the parameter level changes.

**Table 6:** The variance analysis of the elastic modulus of rock-like material.

Variance sources	Seq SS	df	MS	F	p-value	Significant
A	12.92706	4	3.23177	2.89489	0.0484	Yes
B	4.17142	4	1.04286	0.67101	0.6185	No
C	0.90378	4	0.22595	0.13155	0.9685	No
D	13.95826	4	3.48957	3.27717	0.0323	Yes
Error	35.25446	20				

### Poisson’s ratio sensitivity analysis

**Figure 8:**
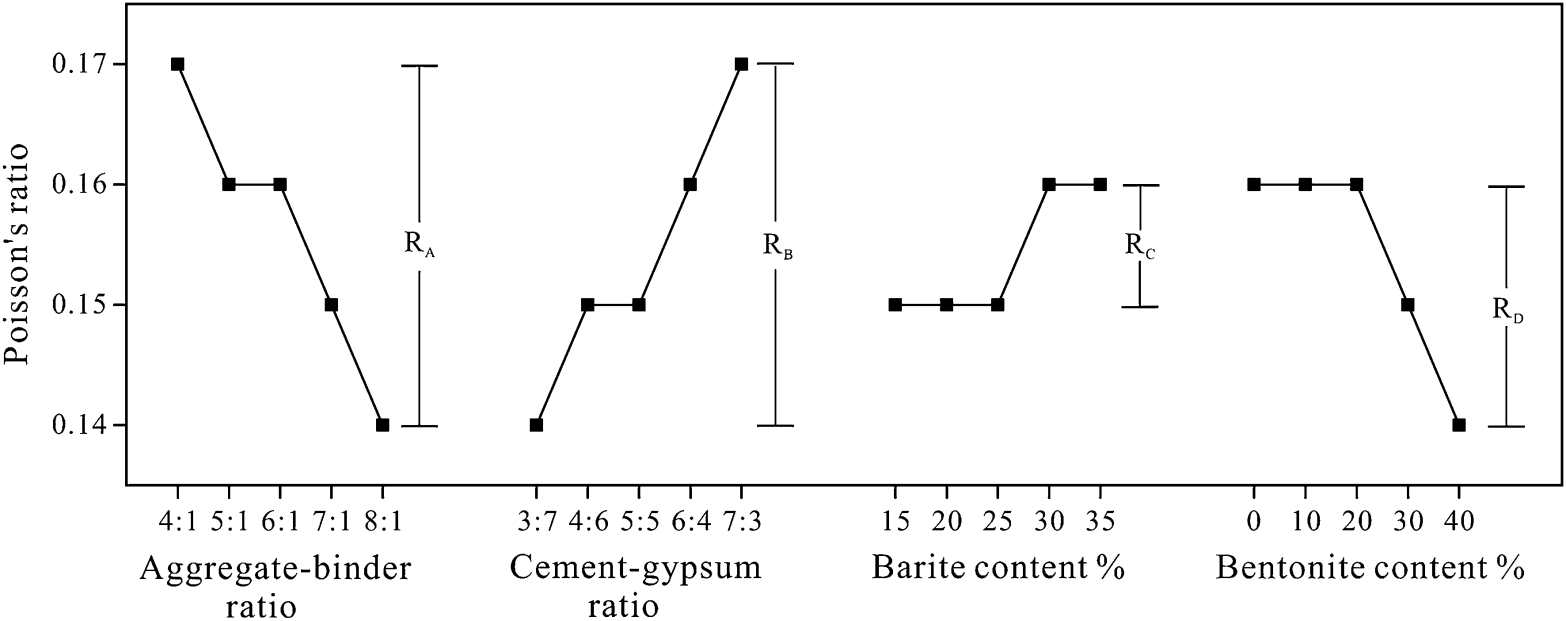
Sensitivity analysis of the Poisson’s ratio.

Figure 8 depicts the sensitivity analysis of different factors on Poisson's ratio. As the parameter level changes, the R-value indicates that the Poisson's ratio fluctuates within a narrow range. Figure 8 demonstrates that the Poisson’s ratio increases significantly with an increase in the cement-gypsum ratio and a decrease in the bentonite content, while other factors have little effect.

The variance analysis of Poisson’s ratio is displayed in Table 7. All p-values are greater than 0.05, indicating that none of the factors had a statistically significant impact on the attributes of rock-like materials, which is consistent with the findings of the sensitivity analysis.

**Table 7:** The variance analysis of the Poisson’s ratio of rock-like material.

Variance sources	Seq SS	df	MS	F	p-value	Significant
A	0.0025	4	0.00064	2.540	0.0718	No
B	0.0021	4	0.00052	1.895	0.1508	No
C	0.0008	4	0.00021	0.618	0.6550	No
D	0.0011	4	0.00028	0.857	0.5065	No
Error	0.0075	20				

### Tensile strength sensitivity analysis

**Figure 9:**
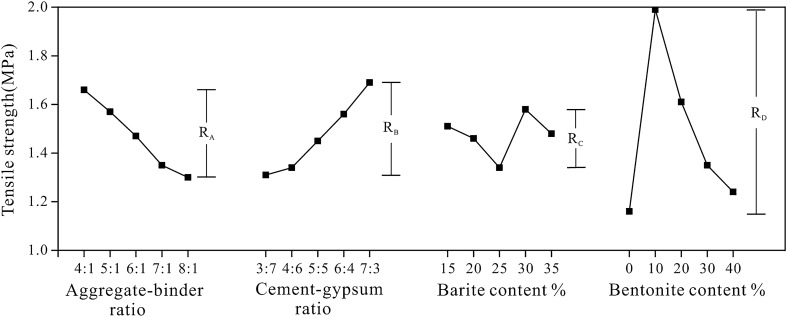
Sensitivity analysis of the tensile strength.

Figure 9 depicts the sensitivity analysis of different factors on tensile strength. According to the R-value, the most sensitive factor is the bentonite content, which increased from 10 to 40%, resulting in a 37.69% reduction in the tensile strength of rock-like materials. Other factors have a similar degree of influence, demonstrating that the bentonite content plays a significant role in determining the tensile strength of rock-like materials. Figure 9 demonstrates that the tensile strength reduces dramatically with an increase in aggregate-binder ratio and a decrease in cement-gypsum ratio, while the tensile strength initially increases and subsequently decreases with an increase in bentonite content. This is because the primary component of bentonite, montmorillonite, has a multi-cracked structure^37^. During curing, microcracks will form within the sample, increasing its porosity and decreasing its strength relative to rock-like materials.

Tensile strength variance analysis is displayed in Table 8. Consistent with the results of the sensitivity analysis, the variance analysis indicates that the bentonite content has a significant effect on the tensile strength of rock-like materials, whereas other factors are unimportant when the parameter level changes.

**Table 8:** The variance analysis of the tensile strength of rock-like material.

Variance sources	Seq SS	df	MS	F	p-value	Significant
A	0.4473	4	0.11183	0.732	0.5810	No
B	0.5169	4	0.12924	0.865	0.5017	No
C	0.1545	4	0.03862	0.231	0.9179	No
D	2.2370	4	0.55926	8.827	0.0003	Yes
Error	3.5042	20				

### Internal friction angle sensitivity analysis

**Figure 10:**
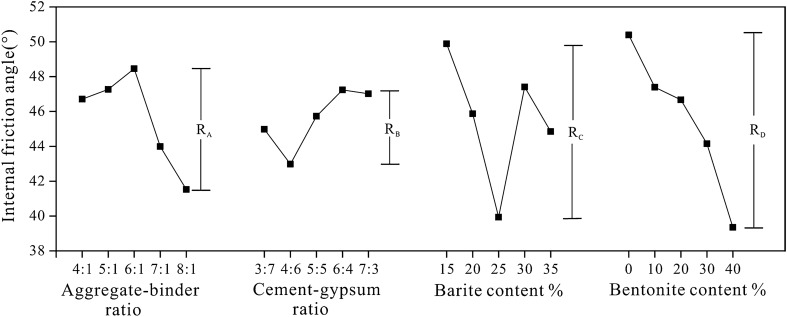
Sensitivity analysis of the internal friction angle.

Figure 10 depicts the sensitivity analysis of various factors on internal friction angle. According to R-value, the most sensitive factor to internal friction angle is the bentonite content. As it varied from 0 to 40%, the internal friction angle of rock-like material reduced by 21.91%. Other factors show a comparable degree of influence, indicating that the bentonite content plays a significant role in determining the internal friction angle of rock-like materials. As demonstrated in Fig. 10, the internal friction angle decreases dramatically as the bentonite content rises, while other variables have little effect.

The variance analysis of the internal friction angle is displayed in Table 9. All p-values are greater than 0.05, indicating that as the parameter level changes, all factors are negligible.

**Table 9:** The variance analysis of the internal friction angle of rock-like material.

Variance sources	Seq SS	df	MS	F	p-value	Significant
A	156.7416	4	39.18539	0.688	0.6088	No
B	59.8350	4	14.95875	0.242	0.9111	No
C	272.3610	4	68.09024	1.330	0.2931	No
D	342.0411	4	85.51029	1.793	0.1700	No
Error	1296.0961	20				

### Cohesion sensitivity analysis

**Figure 11:**
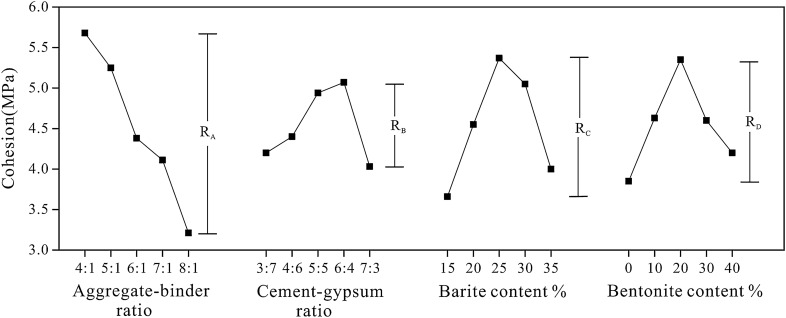
Extremum difference analysis of cohesion.

Figure 11 depicts the sensitivity analysis of various internal friction angle factors. According to the R-value, the most sensitive factor is the aggregate-binder ratio, which rose from 4:1 to 8:1 and decreased the cohesion of rock-like materials by 43.49%. Other factors have a comparable degree of impact, demonstrating that the aggregate-binder ratio is the most important element in determining the cohesion of rock-like materials. Figure 11 demonstrates that the cohesion reduces dramatically as the aggregate-binder ratio increases, whereas other factors have minimal effect. The primary explanation is that when the aggregate-binder ratio grows, the amount of large-sized quartz sand increases, which raises the contact surface roughness and reduces the cohesion of the sample.

Table 10 shows the cohesion variance analysis. Consistent with the results of the sensitivity analysis, the variance analysis indicates that the aggregate-binder ratio has a substantial effect on the cohesion of rock-like materials, whereas other factors are unimportant as the parameter level changes.

**Table 10:** The variance analysis of the cohesion of rock-like material.

Variance sources	Seq SS	df	MS	F	p-value	Significant
A	18.9232	4	4.73080	3.103	0.0387	Yes
B	4.1836	4	1.04591	0.462	0.7624	No
C	10.0808	4	2.52021	1.281	0.3105	No
D	6.2446	4	1.56116	0.723	0.5863	No
Error	49.4196	20				

### Disintegration analysis

**Figure 12:**
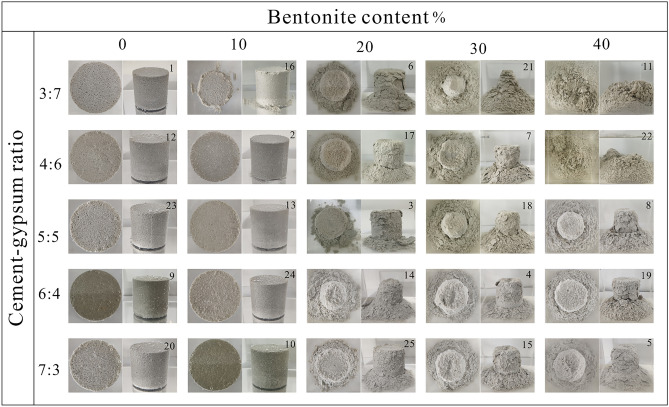
Disintegration experiment of rock-like material samples.

Disintegration is an important characteristic that reflects the hydraulic properties of rocks. Rocks disintegrate into a variety of fragments, including uniform detrital, granular, mud-like, and broken pieces^38^. Here the study focus on whether the sample disintegration is closely related to the mineral composition, particle size composition, and cementation form of the sample. To study the disintegration properties of rock-like materials, a φ50 × 50 mm cylindrical sample was immersed in clear water-filled transparent glassware to carry out the soaking experiment. The soaking time in the experiment scheme was set to 4 h following a thorough evaluation of the total duration of the experiment and the range of changes in residual body mass. Figure 12 depicts the final disintegration findings for each group. The degree of disintegration is categorized into five categories based on the ratio of the dry mass of the residue to the original mass: zero (0.98, 1.0], weak (0.8, 0.98], moderate (0.5, 0.8], strong (0.1, 0.5], and total disintegration [0, 0.1]. Table 11 outlines the grades for each group.

**Table 11:** Disintegration degree of each sample.

Cement-gypsum ratio	Bentonite content (%)				
	0	10	20	30	40
3:7	Zero (1)	Weak (16)	Moderate (6)	Full (21)	Full (11)
4:6	Zero (12)	Zero (2)	Moderate(17)	Moderate (7)	Full (22)
5:5	Zero (23)	Zero (13)	weak (3)	Moderate(18)	Strong (8)
6:4	Zero (9)	Zero (24)	weak (14)	Moderate (4)	Strong (19)
7:3	Zero (20)	Zero (10)	weak (25)	Moderate (15)	Moderate(5)

For materials whose disintegration degree is 0 or weak, it can be used to simulate rocks with good integrity before and after encountering water, but whose strength diminishes obviously with soaking time, such as sandstone, limestone, etc. For materials with moderate or strong degrees of disintegration, it can be used to simulate rocks that partially disintegrate and significantly lose strength when exposed to water, such as argillaceous sandstone, partial structural rock mass, etc. For materials whose disintegration degree is total disintegration, it can be used to simulate the rocks like mudstone and marl, which are relatively complete before coming into contact with water and quickly disintegrate once they do.

After 4 h of soaking, the samples remained intact, and there was no discernible disintegration in the groups with 0 or 10% bentonite content. However, the degree of disintegration strengthens dramatically as the bentonite content rises. In the groups containing 20% bentonite, the exterior was peeled off, but the interior remained intact. The sample disintegrated rapidly in a short period of time, and the disintegration degree was relatively high in the 30% and 40% bentonite content. This is mostly due to the main mineral component of bentonite is montmorillonite, which has a high water absorption capacity and rapidly expands in volume after absorbing water, causing the sample to disintegrate.

As the ratio of cement-gypsum changes from 7:3 to 3:7 while the bentonite content remains constant, the disintegration degree of rock-like materials tends to increase. This behavior is most noticeable when the bentonite content is 40%. On the one hand, the degree of cementation will be weakened with the decrease in cement content. On the other hand, gypsum has a poor water resistance, and its loose and porous characteristics provide seepage channels for further disintegration. Therefore, as the ratio of cement-gypsum falls, disintegration is enhanced.

## Multiple linear regression analysis

The physico-mechanical characteristics of rock-like materials are simultaneously influenced by multiple factors, and the change of each factor will produce certain fluctuations in the parameters. On the basis of experiment data, multiple linear regression analysis was performed to quantify the relationship between various factors and parameters (Table 3). Assuming that Y is the dependent variable and X_n_ (n = 1, 2, …, m) is the independent variable, then the regression analysis model can be stated as formula (1)^39^:

1 $$Y = a_{1} X_{1} + a_{2} X_{2} + \cdots + a_{m} X_{m} + b,$$

where b is the constant term; a1,a2,…,am are the partial regression coefficients.

Let Y_*k*_(k = 1, 2, …, 7) represent the density, UCS, elastic modulus, Poisson’s ratio, tensile strength, internal friction angle, and cohesion of rock-like material; let X_1_, X_2_, X_3_, and X_4_ represent the aggregate-binder ratio, the cement-gypsum ratio, the barite powder content and the bentonite content that influence the physico-mechanical parameters of rock-like material. Following is how the regression equations were obtained:

2 $$\begin{gathered} Y_{1} = - 0.011 \times X_{1} + 0.014 \times X_{2} - 0.258 \times X_{3} - 0.286 \times X_{4} + 2.235 \hfill \\ Y_{2} = - 2.375 \times X_{1} + 1.913 \times X_{2} + 0.088 \times X_{3} + 4.721 \times X_{4} + 27.970 \hfill \\ Y_{3} = - 1.280 \times X_{1} + 0.533 \times X_{2} + 3.594 \times X_{3} - 18.312 \times X_{4} + 16.682 \hfill \\ Y_{4} = 0.001 \times X_{1} + 0.014 \times X_{2} - 0.015 \times X_{3} - 0.046 \times X_{4} + 0.157 \hfill \\ Y_{5} = - 0.094 \times X_{1} + 0.21 \times X_{2} + 0.121 \times X_{3} - 0.481 \times X_{4} + 1.851 \hfill \\ Y_{6} = - 1.364 \times X_{1} + 1.722 \times X_{2} - 17.116 \times X_{3} - 25.32 \times X_{4} + 61.081 \hfill \\ Y_{7} = = - 0.608 \times X_{1} - 0.072 \times X_{2} + 2.388 \times X_{3} + 0.668 \times X_{4} + 7.531. \hfill \\ \end{gathered}$$

**Figure 13:**
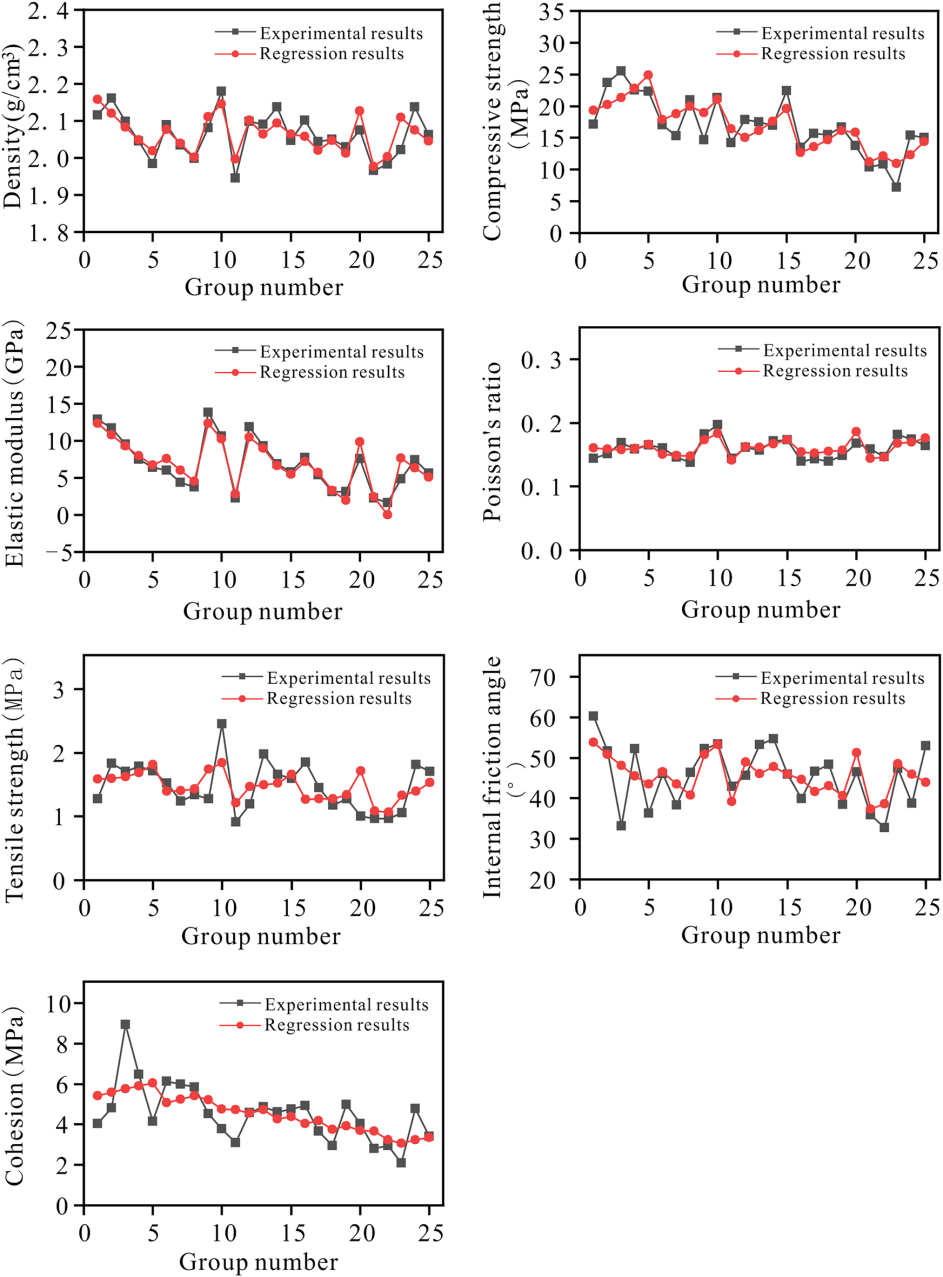
Comparison of the experimental results and regression results of each parameter.

To verify the validity of the result of a regression equation. Using comparative analysis, the difference between the experimental result and the calculation result of the regression equation for each parameter is determined, as illustrated in Fig. 13.

The Fig. 13 demonstrates that the experiment results and regression analysis results for each parameter are in good agreement, indicating that the regression analysis method can be used to construct the quantitative relationship between various factors and the parameters to obtain the parameters of the corresponding rock-like material.

## Application of rock slopes model test

To investigate the water-induced instability mechanism of a rock slope, we established a physical model test using rock-like materials and measured the displacement, and acoustic emission (AE) of the rock slope during the progressive failure process.

### Determine the ratio of rock-like materials

The strength deterioration of the natural rocks is a relatively sluggish process of water–rock interaction, implying that the evolution of a landslide is a lengthy physical and mechanical process. To reconstruct the actual stress state of rock slope in physical model testing, the characteristics of water-induced strength deterioration must be considered by examining the brittle properties of rock-like materials. In previous studies, new rock-like materials with 10% bentonite addition (groups 2, 10, 13, and 24) exhibited a relatively high degree of deterioration with intact specimens, particularly group 24, demonstrating that the addition of bentonite can replicate water-induced strength degradation significantly. Consequently, the ratio of group 24 was selected for the model test, and the corresponding parameters are detailed in Table 3.

### Model test and monitoring program

#### Conceptual geologic model for landslide

**Figure 14:**
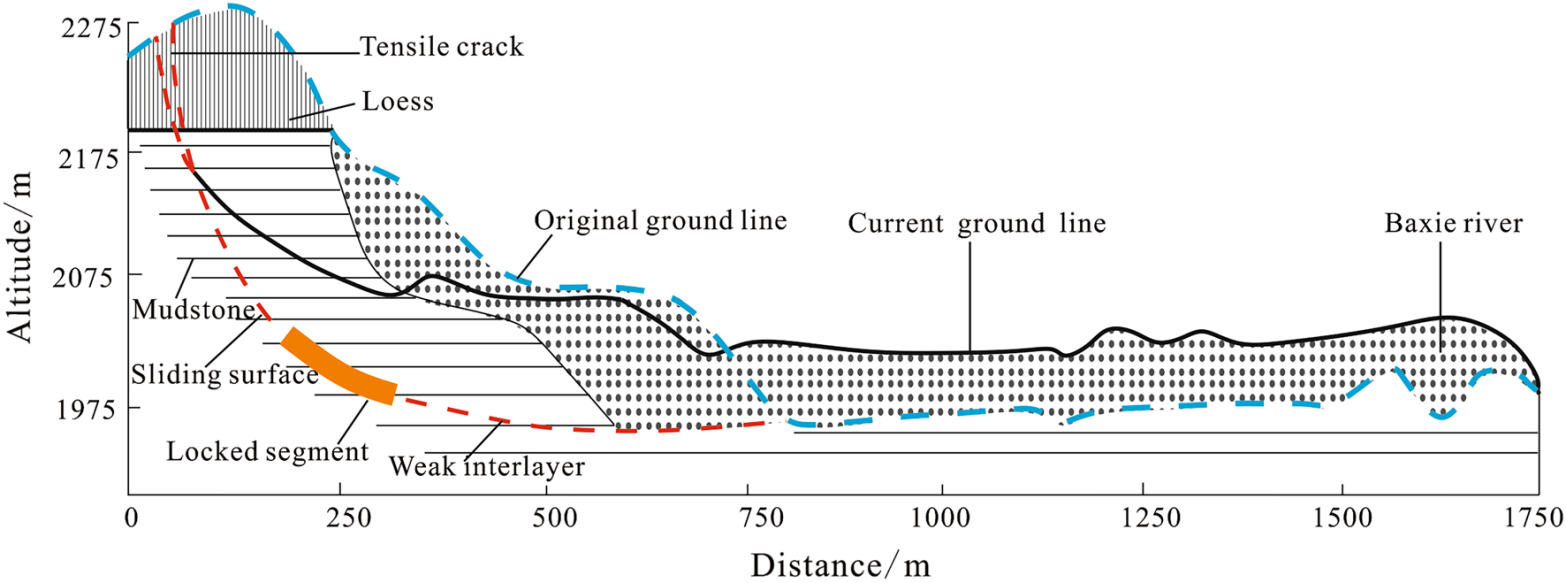
The geological profile of the Saleshan rock slide (modified after Huang et al.^41^).

The Saleshan rock slide happened on March 7, 1983, which destroyed three villages and killed 237 people^40^. The geological profile is shown in Fig. 14. The stability of this form of landslide is controlled by the locked segment in the middle, and there is a weak interlayer on the toe of the slope near horizontal or gently sloping. Under the effect of long-term self-weight stress and continual deterioration of water, the bearing capacity of the locked segment gradually reduced, which led to the downward expansion of the tensile crack of the slope, and finally, the landslide was triggered.

**Figure 15:**
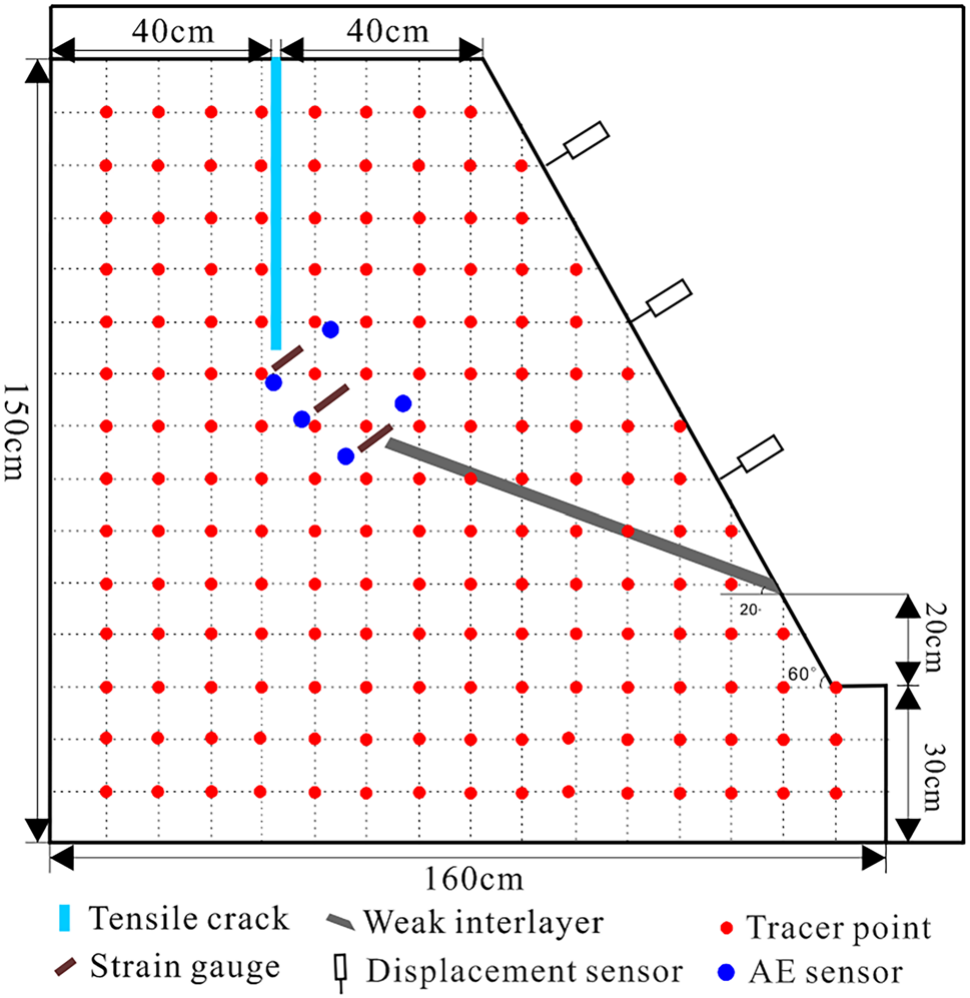
The configuration of the scaled slope model and the monitoring scheme (unit: cm).

To investigate the instability mechanism of this type of landslide under the interaction of water and rock, a scaled rock slide model was established, the size and shape of which are shown in Fig. 15. The post-source tensile crack has a depth of 55 cm, and a thickness of 3 cm. The weak interlayer has a length of 80 cm, a thickness of 3 cm, and an inclination angle of 20°, it is filled with mica powder. The measurement system consisted of strain gauges, AE, and displacement monitoring sensors. Three strain gauges were installed to the locked segment. The front of the slide body was equipped with three displacement monitoring sensors. Five AE sensors were installed around the locked segment.

#### Experimental equipment and process

**Figure 16:**
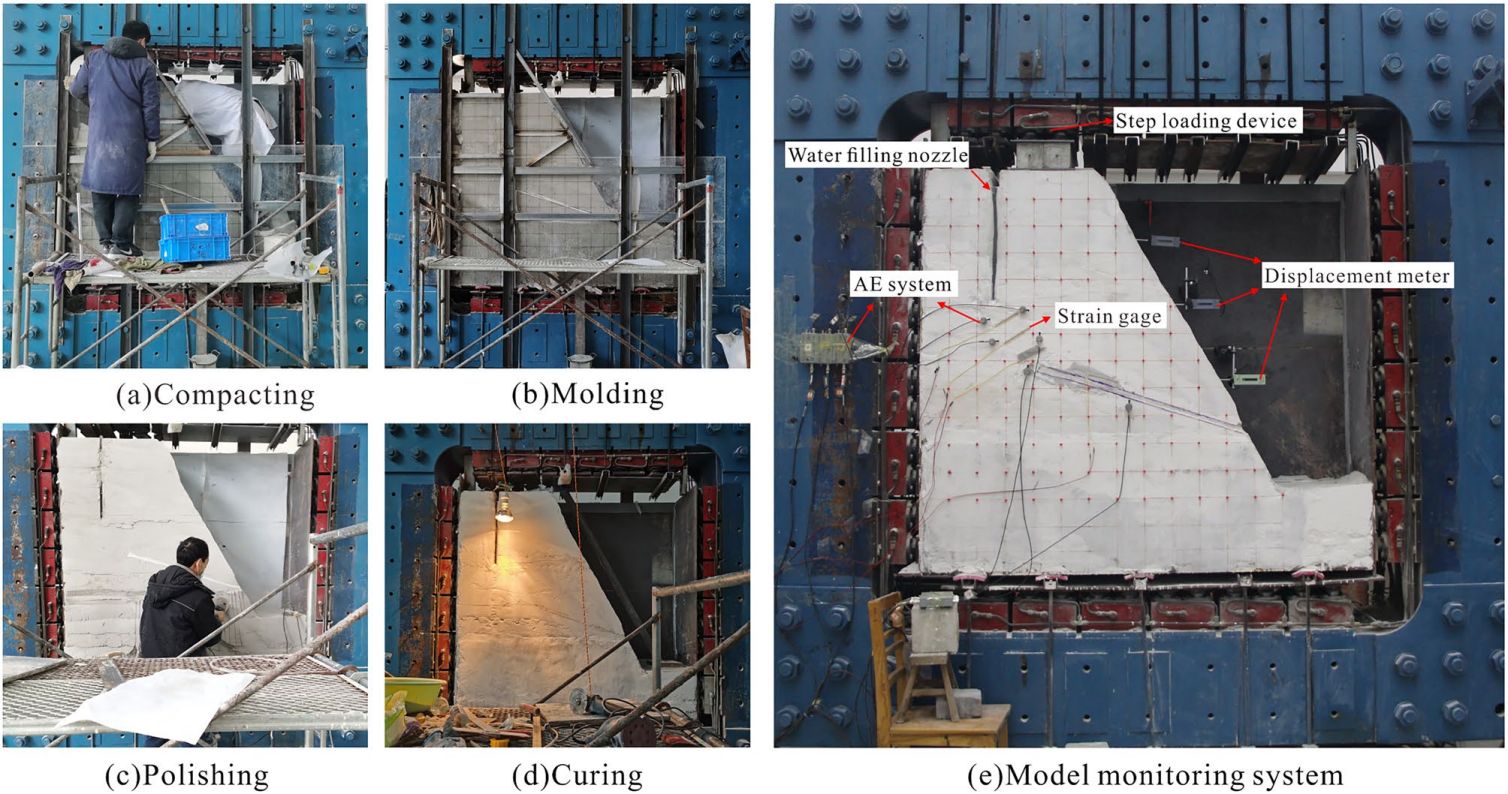
The main production process and monitoring system of the model. (**a**) compacting; (**b**) molding; (**c**) polishing; (**d**) curing; (**e**) model monitoring system.

On the YDM-D geotechnical engineering structural model experimental platform with the maximum model dimensions of 1.6 m × 1.6 m × 0.4 m, the large-scale physical model test was conducted (Fig. 16e). The model was created by compacting (Fig. 16a) and demolded after 48 h of molding (Fig. 16b). After demolding, polished the surface of the model (Fig. 16c) and cured it at room temperature for 30 days to ensure that the interior of the model was fully formed (Fig. 16d). The post-source tensile crack was generated by inserting and then removing a 3 cm steel plate. Finally, waterproof material was applied to waterproof both sides of the crack. Monitoring equipment such as an AE system, strain gauges, and displacement meters were utilized to monitor (supply details on what parameters or properties monitored during the processes of this experiment) the instability process of the slope (Fig. 16e).

#### Test implementation

**Figure 17:**
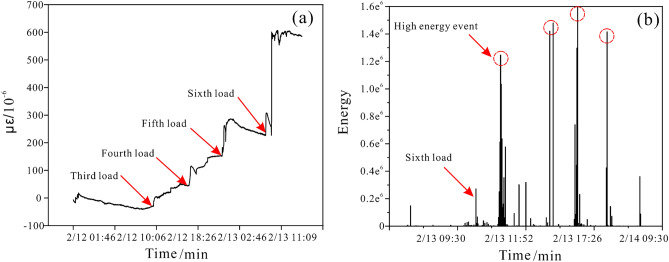
The identification index of water injection time node. (**a**) the strain of the locked segment versus time; (**b**) the AE energy of the locked segment versus time.

Because the water-induced deterioration of rock strength is an extremely slow mechanical process, even if new rock-like materials can significantly accelerate this process, the experiment will inevitably take a long time to complete. To shorten the period, this experiment adopts the method of loading first and then injecting water to promote the damage of the model slope. First, the upper loading device applied the load in stages to approximately 90% of the long-term strength of the material, and then the load was maintained. At this stage, water was injected into the post-source tensile crack, and the water degraded the strength of the middle locked segment. Eventually, the slope model will evolve towards instability under its own weight. The time node of water injection was determined based on strain and acoustic emission data. When the strain data increased greatly (Fig. 17a) or when the AE data produced multiple high-level events (Fig. 17b), which could be determined as the water injection critical node.

### Results

#### Failure evolution

**Figure 18:**
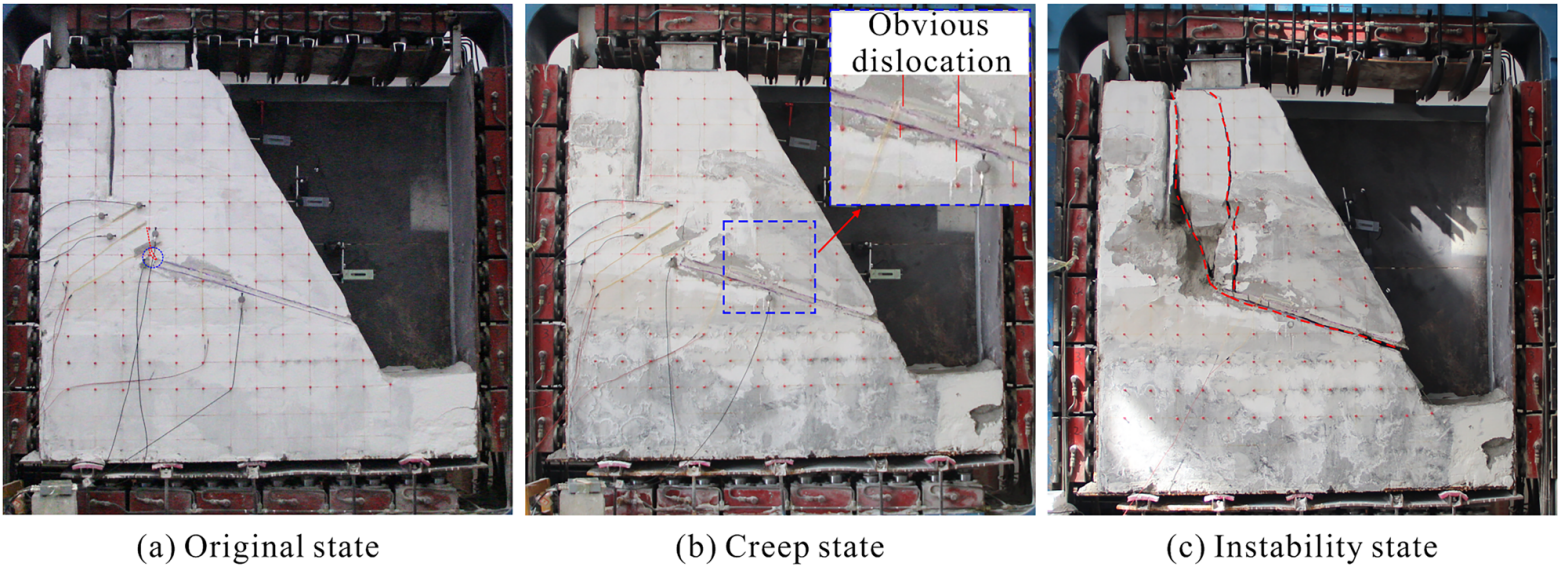
The failure evolution of model slope. (**a**) original state; (**b**) creep state; (**c**) instability state.

Figure 18 reveals the whole failure process of the model slope. Initially, once the upper loading had stabilized, a few tiny fractures formed at the intersection of the locked segment and the post-source tensile crack (Fig. 18a). This is due to the presence of a huge stress concentration at the top of the locked segment, which caused the tensile crack to gradually develop downwards. Consequently, the bearing capacity of the middle locked segment was diminished. With the injection of water until the rock mass in the locked segment was gradually saturated, the sliding body began to generate considerable dislocations along the direction of the weak interlayer (Fig. 18b). This is because the existence of water increases the sliding force and, more crucially, the water–rock effect accelerates the deterioration of the locked segment. After 16 days of continuous water injection, the locked segment was finally entirely sheared, and the landslide was triggered (Fig. 18c). Before the landslide is triggered, a tremendous noise can be clearly heard, which is generated by the complete penetration of the locked segment. This phenomenon has been observed in numerous rock landslides^42,43^.

#### Displacement of the sliding body

**Figure 19:**
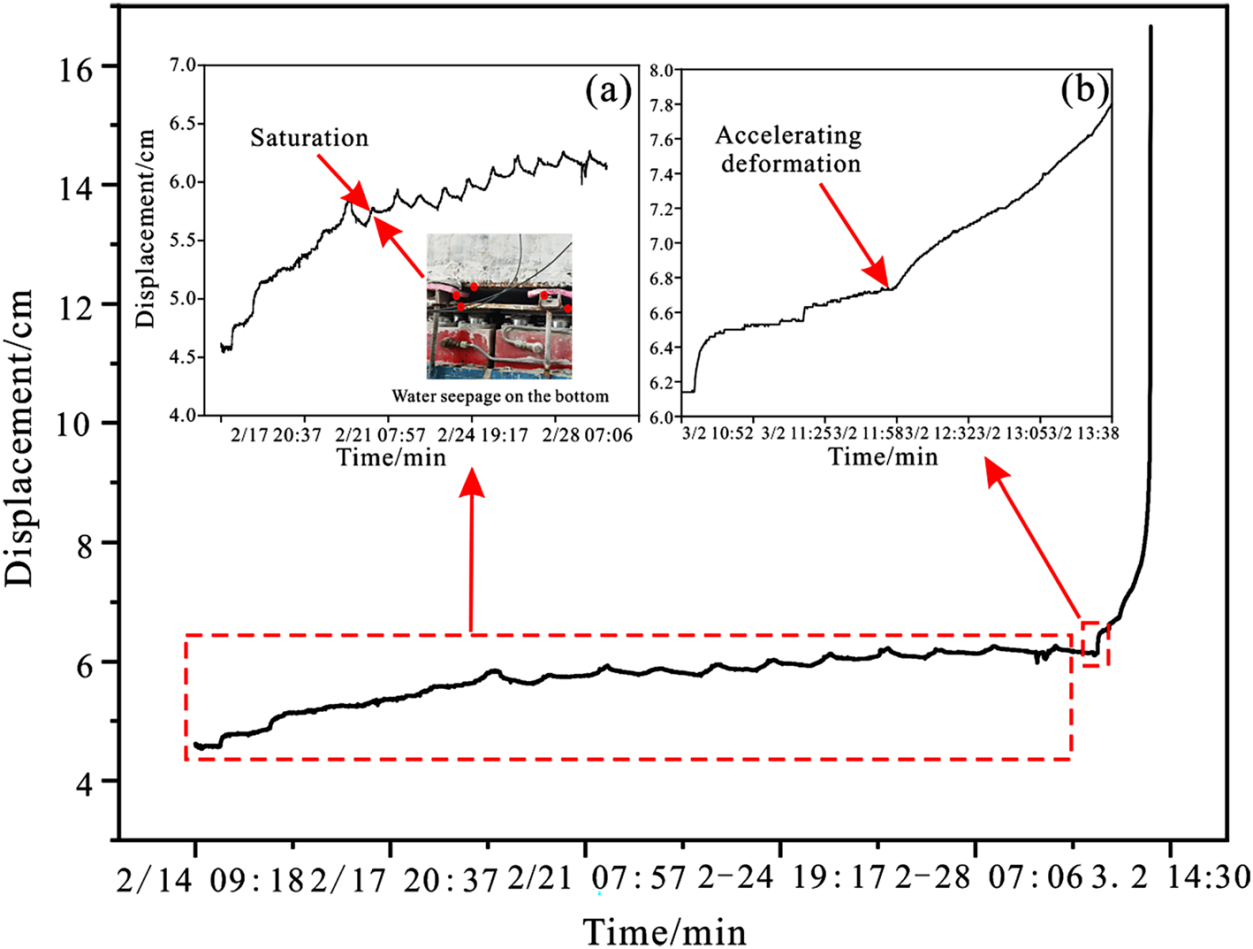
The displacement of the sliding body versus time. (**a**) Partial enlargement of creep state; (**b**) partial enlargement of accelerated deformation state.

The evolution law of displacement data obtained by the displacement meter in the middle of the slope is shown in Fig. 19. As depicted in Fig. 19a, the displacement of the slope grew significantly following the start of water injection. This occurs because the strength of the locked segment in the middle of the slope degrades as it comes into contact with water, and the anti-sliding force it provides reduces, causing the slope to advance. With continued water injection, the locked segment progressively becomes saturated, and the rate of displacement growth begins to decelerate. After 16 days of continuous water injection, the cumulative damage of the locked segment reached its peak, resulting in the rapid expansion of internal cracks, and then the slope entered the stage of accelerated deformation (Fig. 19b), indicating that the displacement growth rate increased significantly.

Through the aforementioned analysis, it is not difficult to conclude that the new rock-like material can accurately duplicate the fracture mechanism of a rock slope under the interaction between water and rock, as well as the evolution law of slope instability. Similarly, this type of material is also suitable for other similar rock engineering studies.

## Conclusions

In this study, the physico-mechanical and disintegrating properties of hard rock-like materials under different material mixture proportions were studied. Based on the outcomes of the experiment, the following conclusions can be drawn:

1. A new rock-like material composed by barite powder, quartz sand, bentonite, cement, and gypsum promote rock strength deterioration during water–rock interaction. This novel rock-like material features a high volumetric weight, water sensitivity, and a simple preparation process. The physico-mechanical properties of rock-like materials have a broad distribution range, which allows them to meet the needs of various types of rocks.
2. The bentonite content significantly affects the density, elastic modulus, and tensile strength of rock-like materials, whereas the aggregate-binder ratio significantly affects the uniaxial compressive strength, elastic modulus, and cohesion of rock-like materials.
3. The disintegration experiment demonstrates that the bentonite content and the cement-gypsum ratio are important factors that affect the disintegration of rock-like materials, with the bentonite content being the more relevant factor. Therefore, the bentonite content and cement-gypsum ratio should be emphatically considered in the process of selecting rock-like materials that simulate the characteristics of water-induced strength degradation.
4. On the basis of the orthogonal test results, regression equations between influencing factors and physico-mechanical properties were derived, which can be used to estimate the physico-mechanical parameters and thus select suitable materials for physical model tests.
5. After application analysis, it is confirmed that the rock-like material produced for this study is applicable to large-scale physical model tests of rock landslides, and that the failure mode is consistent with actual engineering. In addition, it has a high application value and can be utilized in tunnel excavation and coal mining research.

## Data Availability

All data that support the findings of this study are available from the corresponding author upon reasonable request.

## References

[1] Huang RQ (2009). Some catastrophic landslides since the twentieth century in the southwest of China. Landslides.

[2] Gao KP, Xu ZX, Mwalupaso GE, Cheng ZY, Wang YT (2022). A numerical study on the strata movement induced by mining under a jointed-rock slope. Geotech. Res..

[3] Wang HL, Xu WY, Shao JF (2014). Experimental researches on hydro-mechanical properties of altered rock under confining pressures. Rock Mech. Rock Eng..

[4] Raj M, Sengupta A (2014). Rain-triggered slope failure of the railway embankment at Malda, India. Acta Geotech..

[5] Senthilkumar V, Chandrasekaran SS, Maji VB (2018). Rainfall-induced landslides: Case study of the Marappalam landslide, Nilgiris district, Tamil Nadu, India. Int. J. Geomech..

[6] Yang H, Yang T, Zhang S, Zhao F, Hu K, Jiang Y (2020). Rainfall-induced landslides and debris flows in Mengdong Town, Yunnan Province, China. Landslides.

[7] Yu LC (2021). Analysis of the mechanism and failure mode of landslides subjected to transient seepage in a Piedmont region of Nanjing area. Bull. Eng. Geol. Environ..

[8] Xue L, Qin SQ, Li P, Li GL, Oyediran IA, Pan XH (2014). New quantitative displacement criteria for slope deformation process: From the onset of the accelerating creep to brittle rupture and final failure. Eng. Geol..

[9] Chen HR, Qin SQ, Xue L, Yang BC, Zhang K (2018). A physical model predicting instability of rock slopes with locked segments along a potential slip surface. Eng. Geol..

[10] Wang KL, Lin ML (2011). Initiation and displacement of landslide induced by earthquake—A study of shaking table model slope test. Eng. Geol..

[11] Li C (2016). Model test on rainfall-induced loess–mudstone interfacial landslides in Qingshuihe, China. Environ. Earth Sci..

[12] He CC, Hu XL, Tannant DD, Tan FL, Zhang YM, Zhang H (2018). Response of a landslide to reservoir impoundment in model tests. Eng. Geol..

[13] Cogan J, Gratchev I (2019). A study on the effect of rainfall and slope characteristics on landslide initiation by means of flume tests. Landslides.

[14] Fan G, Zhang JJ, Wu JB, Yan KM (2016). Dynamic response and dynamic failure mode of a weak intercalated rock slope using a shaking table. Rock Mech. Rock Eng..

[15] Xu ZL, Luo YB, Chen JX, Su ZM, Zhu TT, Yuan JP (2021). Mechanical properties and reasonable proportioning of similar materials in physical model test of tunnel lining cracking. Constr. Build. Mater..

[16] Fumagalli E (1973). Statical and Geomechanical Model.

[17] Han BL, Chen XL, Song YL, Li HM (1997). Research on similar material of rockmass. Eng. J. Wuhan Univ..

[18] Kim SH, Burd HJ, Milligan GWE (1998). Model testing of closely spaced tunnels in clay. Géotechnique.

[19] Zuo BC, Chen CX, Liu CH, Shen Q, Xiao GF, Liu XW (2004). Research on similar material of slope simulation experiment. Rock Soil Mech..

[20] Yang GX, Qi SW, Wu FQ, Zhan ZF (2017). Seismic amplification of the anti-dip rock slope and deformation characteristics: A large-scale shaking table test. Soil Dyn. Earthq. Eng..

[21] Zhang Y, Cao Z, Shi XM (2021). Study on the water-physical properties of the cement-plaster bonded rock-like materials. Adv. Civ. Eng..

[22] Wang HP, Li SC, Zhang QY, Li Y, Guo XH (2006). Development of a new geomechanical similar material. Chin. J. Rock Mech. Eng..

[23] Dong JY, Yang JH, Yang GX, Wu FQ, Liu HS (2012). Research on similar material proportioning test of model test based on orthogonal design. J. China Coal Soc..

[24] Dong JY, Wang C, Huang ZQ, Yang JH, Xue L (2021). Dynamic response characteristics and instability criteria of a slope with a middle locked segment. Soil Dyn. Earthq. Eng..

[25] Dong ML, Zhang FM, Lv JQ, Hu MJ, Li ZN (2020). Study on deformation and failure law of soft-hard rock interbedding toppling slope base on similar test. Bull. Eng. Geol. Environ..

[26] Cui GJ, Zhou CY, Liu Z, Xia C, Zhang LH (2022). The synthesis of soft rocks based on physical and mechanical properties of red mudstone. Int. J. Rock Mech. Min..

[27] Liu HD, Liu JJ, Zhang SY, Feng LY, Qi L (2022). Stress-strain and acoustic emission characteristics of cement-based materials used to simulate soft rock with fractures. Sci. Rep..

[28] Wang YS, Zhao B, Li J (2017). Mechanism of the catastrophic June 2017 landslide at Xinmo Village, Songping River, Sichuan Province, China. Landslides.

[29] Huang D, Zhong Z, Gu DM (2019). Experimental investigation on the failure mechanism of a rock landslide controlled by a steep-gentle discontinuity pair. J. Mt. Sci..

[30] Chen GQ, Li L, Zhao C, Huang RQ, Guo F (2016). Acceleration characteristics of a rock slide using the particle image velocimetry technique. J Sens..

[31] Fan W, Wei YN, Deng L (2018). Failure modes and mechanisms of shallow debris landslides using an artificial rainfall model experiment on Qin-ba mountain. Int. J. Geomech..

[32] Liu HD, Li DD, Wang ZF, Geng Z, Li LD (2019). Physical modeling on failure mechanism of locked-segment landslides triggered by heavy precipitation. Landslides.

[33] Ge Z, Gao Z, Sun R, Zheng L (2012). Mix design of concrete with recycled clay-brick-powder using the orthogonal design method. Constr. Build. Mater..

[34] Xu C, Cui Y, Xue L, Chen HR, Dong JY, Zhao HX (2023). Experimental study on mechanical properties and failure behaviours of new materials for modeling rock bridges. J. Market. Res..

[35] Ulusay R (2014). The ISRM Suggested Methods for Rock Characterization Testing and Monitoring.

[36] Namkon L (2018). Experimental design of a well cement slurry for rapid gel strength development. Constr. Build. Mater..

[37] Fayza BG, Agnès S, Jean-Pierre B (2006). Montmorillonite based artificial nacre prepared via a drying process. Mat. Sci. Eng. B Adv..

[38] Zhang Z, Gao WH (2020). Effect of different test methods on the disintegration behaviour of soft rock and the evolution model of disintegration breakage under cyclic wetting and drying. Eng. Geol..

[39] Jin RY, Chen Q, Soboyejo ABO (2018). Non-linear and mixed regression models in predicting sustainable concrete strength. Constr. Build. Mater..

[40] Kang C, Zhang F, Pan F, Peng J, Wu W (2018). Characteristics and dynamic runout analyses of 1983 Saleshan landslide. Eng. Geol..

[41] Huang RQ, Chen GQ, Guo F, Zhang GF, Zhang Y (2016). Experimental study on the brittle failure of the locking section in a large-scale rock slide. Landslides.

[42] Walter M, Arnhardt C, Joswig M (2012). Seismic monitoring of rockfalls, slide quakes, and fissure development at the Super-Sauze mudslide, French Alps. Eng. Geol..

[43] Chen TC, Lin ML, Wang KL (2014). Landslide seismic signal recognition and mobility for an earthquake-induced rockslide in Tsaoling, Taiwan. Eng. Geol..

